# Artificial tactile and proprioceptive feedback improves performance and confidence on object identification tasks

**DOI:** 10.1371/journal.pone.0207659

**Published:** 2018-12-05

**Authors:** Matthew A. Schiefer, Emily L. Graczyk, Steven M. Sidik, Daniel W. Tan, Dustin J. Tyler

**Affiliations:** 1 Case Western Reserve University, Cleveland, Ohio, United States of America; 2 Advanced Platform Technology (APT) Center, Louis Stokes Cleveland Veterans Affairs Medical Center (LSCVAMC), Cleveland, Ohio, United States of America; 3 Cleveland Functional Electrical Stimulation (FES) Center, LSCVAMC, Cleveland, Ohio, United States of America; Tokai University, JAPAN

## Abstract

Somatosensory feedback of the hand is essential for object identification. Without somatosensory feedback, individuals cannot reliably determine the size or compliance of an object. Electrical nerve stimulation can restore localized tactile and proprioceptive feedback with intensity discrimination capability similar to natural sensation. We hypothesized that adding artificial somatosensation improves object recognition accuracy when using a prosthesis. To test this hypothesis, we provided different forms of sensory feedback–tactile, proprioceptive, or both–to two subjects with upper limb loss. The subjects were asked to identify the size or mechanical compliance of different foam blocks placed in the prosthetic hand while visually and audibly blinded. During trials, we did not inform the subjects of their performance, but did ask them about their confidence in correctly identifying objects. Finally, we recorded applied pressures during object interaction. Subjects were free to use any strategy they chose to examine the objects. Object identification was most accurate with both tactile and proprioceptive feedback. The relative importance of each type of feedback, however, depended on object characteristics and task. Sensory feedback increased subject confidence and was directly correlated with accuracy. Subjects applied less pressure to the objects when they had tactile pressure feedback. Artificial somatosensory feedback improves object recognition and the relative importance of tactile versus proprioceptive feedback depends on the test set. We believe this test battery provides an effective means to assess the impact of sensory restoration and the relative contribution of different forms of feedback (tactile vs. kinesthetic) within the neurorehabilitation field.

## Introduction

Accurate and efficient manipulation of objects with one’s hands requires tactile sensation and proprioception [1,2]. Cutaneous anesthesia reduces precision [3] and the ability to distinguish object compliance [4]. Loss of function and somatosensation after an upper extremity (UE) amputation can be devastating. Advanced UE prostheses have continued to evolve to mimic anthropomorphic hand function. Even if they achieve perfect mechanical mimicry of the normal human hand, the lack of effective somatosensory feedback will significantly limit their function and use.

Sensory substitution methods attempt to replace lost somatosensory input. Tension in the cabling of the harness of body-powered prostheses provides indirect sensory feedback that is correlated to the force exerted by the terminal device [5]. For many amputees, it is the indirect sensory connection to the terminal device that makes the body-powered prosthesis a more attractive prosthetic option than a myoelectric prosthesis. For myoelectric prostheses, sensory substitution with vibratory tactors provides information about the prosthetic hand’s position or applied pressure. These improve control in and outside of the laboratory [6–8]. However, sensory substitution is not widely adopted, perhaps due to the cognitive burden of modal and spatial interpretation.

Electrical stimulation applied through electrodes implanted in or around the residual sensory nerves in the limb can elicit sensation that is perceived to be located on the missing hand, creating a more natural approximation of tactile and proprioceptive feedback. Electrical stimulation produces naturalistic sensations in small, localized areas of the hand that can be reliably scaled in perceived intensity [9–12]. Naturalistic sensation located on the perceived hand should be more intuitive than sensory substitution, since it is more spatially and modally congruent, thereby providing greater functional benefits with less training. In addition, naturalistic sensation could improve prosthesis embodiment [13], thereby reducing prosthesis abandonment.

To determine the impact of sensation on prosthesis functionality, functional tasks that specifically depend on or integrate with sensory feedback must be performed with the prosthesis. However, the standardized functional tests available for upper limb prostheses were not designed for assessing the impact of sensation [14–16]. In the natural hand, object identification tasks are performed to measure sensory capabilities and hand function in persons with carpal tunnel syndrome, neuropathy, or who have had nerve repair surgery [17,18] [19,20]. In these tests of object discrimination with a natural hand, persons are asked to determine object shape, object size, or object texture [17,19]. While objects that differ in size or texture can be differentiated with vision alone, determination of an object’s compliance requires tactile feedback and knowledge of the forward control model during object deformation [4]. Determination of object shape and size relies on knowledge of the hand position (i.e. proprioceptive information) in addition to tactile feedback during manipulation of the object [21,22]. Object discrimination tasks demonstrate how well sensory information is interpreted and utilized, and thus are informative tests to assess the interpretability and usefulness of sensory feedback in UE prostheses.

Prior studies have shown the potential for sensory restoration to improve object identification [11,23]. In this study, we evaluated the ability of amputees to perform a battery of object identification tasks using their prostheses with sensation provided through peripheral nerve stimulation. There were two goals for this study. First, to explore the extent to which different forms of sensation (i.e. tactile vs. proprioceptive) provided through extraneural stimulation could improve object identification with a prosthesis. And second, to evaluate the ability of this test battery to capture the impact of the restored sensation on functional and psychological outcomes. The subjects in this study are upper limb amputees implanted with extraneural cuff electrodes. Through these electrodes, we stimulated the nerve to provide feedback about fingertip pressure and hand aperture. The intensity of the perceived sensation was linearly scaled to the amount of pressure exerted at the prosthetic fingertips or to the degree of hand aperture [10]. Subjects used their own prosthetic hand with externally-attached sensors, their own, prosthetist-fit socket, and their own myoelectric control scheme in this study. These conditions were chosen because we aimed to reproduce prosthesis usage conditions that were as realistic as possible, so that our findings could be applicable to understanding the potential rehabilitation impact of artificial sensory feedback provided through neural stimulation. Our hypothesis is that subjects would perform best when they received information about both fingertip pressure and hand aperture, rather than either no stimulation-elicited feedback or only one form of stimulation-elicited feedback.

## Methods

### Subjects

**Fig 1:**
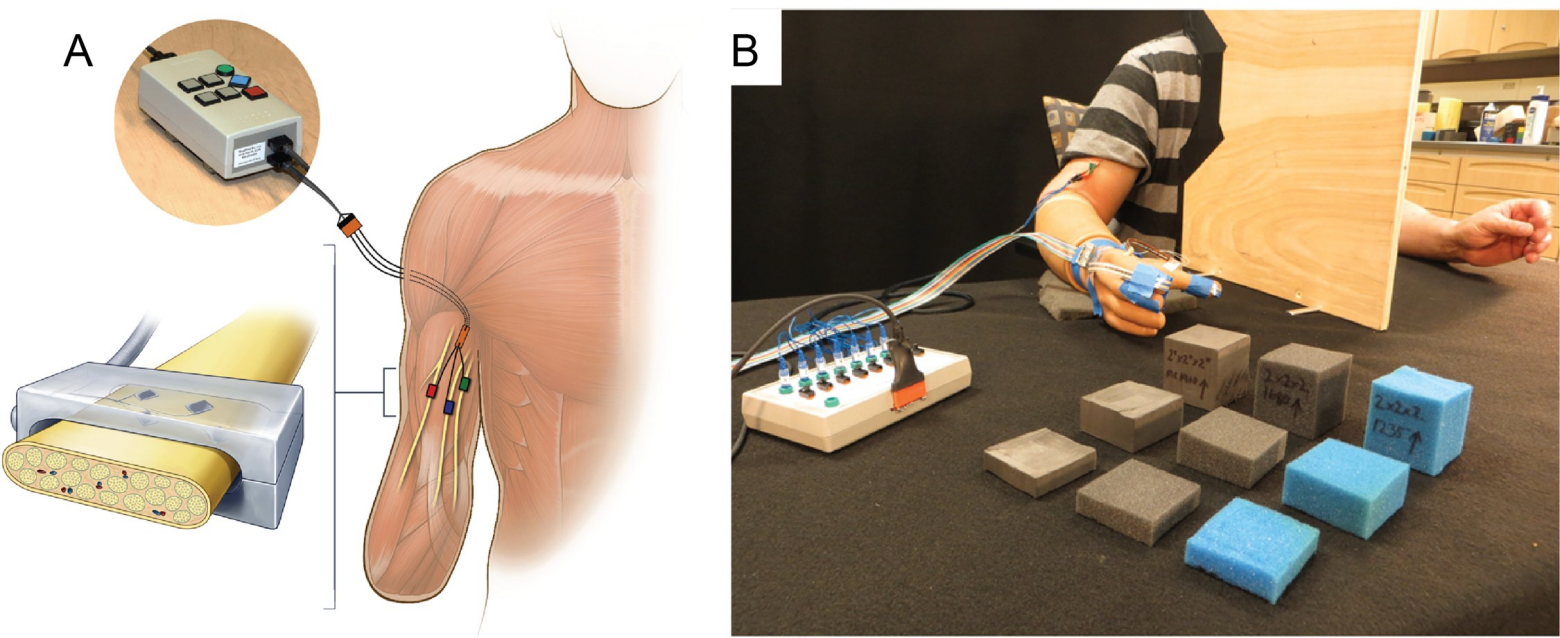
Neural interface and experimental setup. A) Surgeons implanted FINEs around the median, radial, and ulnar nerves of subjects S1 and S2[10]. Surgeons tunneled leads from the cuffs to a percutaneous exit site in the lateral upper arm. The percutaneous leads connected to an external nerve stimulator, which delivered pulses of electrical stimulation to the nerve. S1’s amputation is at the level of the wrist. S2’s amputation is at the proximal forearm. B) Force sensitive resistors were mounted to the subject’s hand prosthesis on the index, thumb, and middle finger. A flex sensor was mounted to the back of the hand to measure prosthetic aperture. The information transduced by these sensors was sent to a computer, which transformed them into appropriate stimulation patterns that were sent to the percutaneous leads. The subjects’ task was to identify either the size or the compliance of a foam block presented to the prosthetic hand, while the subject was blindfolded and wearing noise-cancelling headphones.

To restore sensation, we implanted 8-channel Flat Interface Nerve Electrodes (FINEs) (Ardiem Medical, Indiana, Pennsylvania) around the medial, radial, and ulnar nerves in two subjects, S1 and S2, who have transradial amputations (Fig 1A). Lead wires traveled subcutaneously from the implanted cuffs to percutaneous exit sites on the lateral arm. The outpatient procedures occurred at the Louis Stokes Cleveland Veterans Affairs Medical Center (LSCVAMC) following approval by the Institutional Review Board (IRB) and the FDA under an Investigational Device Exemption (IDE). Written informed consent was obtained from each subject prior to their inclusion in the study. More details are available [9,10,13,24]

### Instrumenting the hand

Subjects used their own prosthetic socket with their standard myoelectric control settings for a standard agonist/antagonist myoelectric controller. We instrumented an Ottobock SensorHand Speed prosthetic hand with low-profile force sensitive resistors (FSRs; Tekscan FlexiForce A201) mounted on the pads of the thumb, index finger, and middle finger of the prosthesis (Figs 1 and S1). A flex sensor (Abrams Gentile Entertainment) measured the hand aperture, i.e. the opening span of the hand. We calibrated the FSRs with known weights. We calibrated the flex sensor by having the subject close the prosthetic hand to fixed apertures. The hand’s built-in slip sensor was disabled.

### Electrical stimulation

The experiments occurred between 524–666 and 155–574 days post-implant for S1 and S2, respectively. We delivered biphasic, charge-balanced, cathode-first electrical stimulation to four channels of the subjects’ median nerve cuff via an external, programmable stimulator connected to the percutaneous leads. For each electrode contact, we found the sensory threshold using a published method [10,13].

To convey tactile sensations via stimulation, we utilized the sinusoidal stimulation paradigm described previously [9]. We found in that study, that pulse trains with small sinusoidal modulations in PW, typically over a range of 5–10 μs, evoked percepts that were described as “pressure” more frequently than other stimulation patterns. In the present study, we utilize this small, 5–10 μs sinusoidal PW modulation stimulation for the tactile percepts. We modulated the PW using a 1 Hz sinusoidal envelope to produce a sense of pressure (10). The stimulation produced sensation on the thumb, index, and middle finger pads of the missing hand (S1B Fig) [9,10].

In S1, a fourth channel produced tactile sensation on the thenar eminence of the missing hand as a substitution for proprioception because S1 does not perceive kinesthesia. In S2, the fourth contact produced a proprioceptive sensation of middle finger flexion. To convey aperture information, the stimulation pulse train consisted of pulses in which the pulse width was varied sinusoidally, again at 1 Hz, but the modulation covered a larger range, up to 150 microseconds. This stimulation paradigm was used to convey aperture information regardless of whether the percept was tactile (for S1) or proprioceptive (for S2) in modality.

Sensations associated with the pressure sensors for both subjects and with the aperture sensor for S1 were tactile in modality and were described using words such as “pressure,” “tingle,”, “vibration,” and “touch” [9,10]. The sensation associated with the aperture sensor for S2 was described qualitatively as “contraction,” “movement,” and “pulling.”

The FSRs on the prosthesis fingertips modulated the stimulus delivered to the contacts in the FINE that elicited tactile sensation on the thumb, index, and middle finger pads. As force applied to an FSR increased, the stimulation pulse frequency (PF) on the channel associated with that sensor increased in a linearly proportional manner between 10 and 125 Hz. The flex sensor similarly regulated the stimulation PF of its associated channel as the hand closed, with more hand closure corresponding to higher stimulation PF. Stimulation did not interfere with the subjects’ myoelectric control of the prosthesis.

### Functional tests

We administered a 3-object, forced-choice object identification test (OIT) to assess the effect of sensory feedback on object discrimination performance. During each test, the subject was blindfolded and wore noise-cancelling headphones. Removing visual input addressed the desire of most amputees to not require constant visual attention on the object. Removing auditory input prevented the subject hearing the prosthesis motor. We presented a foam block between the thumb and index finger of the prosthesis. The subject was asked to identify the size or the compliance of the block, selecting from three block heights– 1.3 (small), 2.5 (medium), and 5.1 (large) cm (0.5, 1.0 and 2.0 inches)–or three block compliances–“soft,” “medium”, and “hard,” corresponding to blocks made of 1235 polyether foam, 1680 polyether foam, and 1900 cross-linked polyethylene foam, respectively (Fig 1B). The “soft”, “medium”, and “hard” blocks required 1.0, 5.4, and 9.3 N to compress 1 mm, respectively. All blocks were identical in length and width, which measured approximately 5.1 cm (2 inches). When referring to a block throughout this paper, the nomenclature will always be compressibility followed by size, such as “soft, small block.”

Each experimental trial set consisted of 15 trials, and the subject was instructed whether they would identify block size or block compliance during the trial set prior to starting the task. We administered the OIT under 4 conditions: (1) without sensory feedback; (2) with pressure feedback only; (3) with hand aperture feedback only; (4) with both pressure and aperture feedback. Prior to each trial set, we told the subject which feedback he would be given, and the subject could manipulate the foam blocks with the prosthesis and specified sensory feedback condition until he felt familiar with the blocks and comfortable with the sensory feedback provided. The subject was then blindfolded and required to wear noise-cancelling headphones that played white noise. We fixed either the block size or compliance while randomly presenting blocks with the three variations of the other attribute. Thus, the probability of correct block identification due to random chance was 33.3%. After positioning the foam block between the thumb and index of the fully opened prosthetic hand, we signaled the subject to close his hand by tapping his shoulder. There was no time limit and subjects were not instructed on what strategy to use when making their decisions. A trial set consisted of 15 randomized presentations with each block presented 5 times.

For each trial set, we asked the subject to rate his confidence in his ability to accurately identify the blocks in the upcoming set, on a scale of 0% to 100%, which was then plotted versus accuracy. The subject also described the strategy he employed to identify the blocks. We recorded the pressure applied to the foam block during each trial, but did not instruct the subject on how much force to apply to a block nor how to make decisions about object identification, and we were blinded to the amount of force the subject applied to a block during the experiments. Subjects were not instructed by the experimenters on how to optimally perform the tasks, but rather self-selected strategies for all tests.

### Statistical analyses

We created confusion matrices of presented block versus subject response for each stimulus condition and applied a χ2 test to determine if responses were randomly distributed [23]. We also used an ANOVA with a Bonferroni correction for multiple comparisons to assess the effect of feedback type on 1) the proportion of blocks that the subject correctly identified and 2) the subject’s confidence. We fit binary logistic equations to the proportion of correct identifications and confidence data as a function of the experiment and feedback type, accounting for interactions. We analyzed the data from trials where we asked the subject to 1) identify block size and 2) identify block compliance. Results obtained during control experiments during where there was no stimulus feedback were compared against chance (33.33%) using a one-sample test of proportion.

We also analyzed the pressure exerted by the subject on the foam block. We censored forces exceeding 4.4 N because they exceeded the FSR’s linear range. We used the VGAM package in R (The R Foundation for Statistical Computing, Vienna, Austria) to fit a right-censored linear model. We fit a separate model for each subject that included the main effects of feedback type, block size and hardness, and experimental date, as well as their interactions. We used the average of the pressure sensors as the fitted response variable. Because only one subject perceived kinesthesia, we analyzed subjects independently except when otherwise stated. In all cases, p ≤ 0.05 was considered significant.

## Results

S1 evaluated 903 blocks during 6 experimental sessions spanning 142 days. S2 evaluated 1550 blocks during 6 experimental sessions spanning 419 days. Performance was not affected by time (ANOVA; S1: p = 0.105, S2: p = 0.585).

### Block identification accuracy

**Fig 2:**
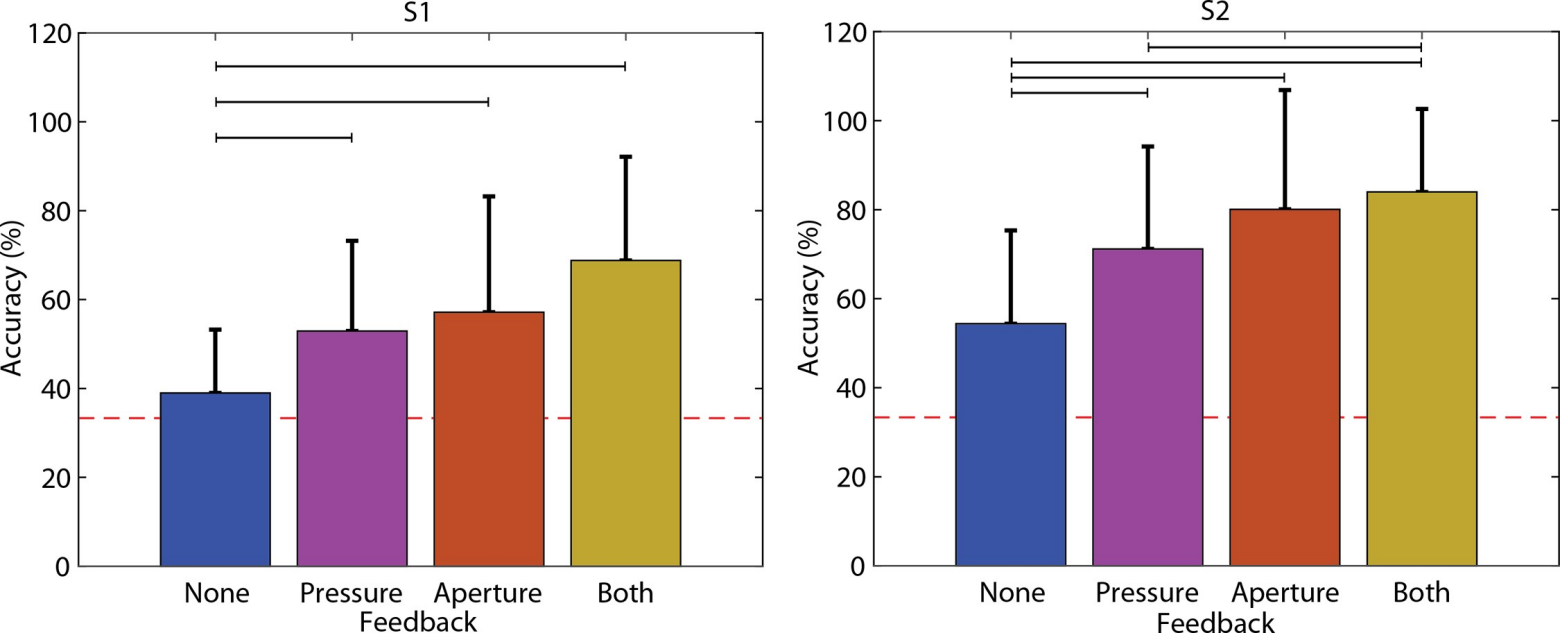
Average and standard deviation of accuracy of foam block identification for both subjects as a function of feedback. The red dashed line is chance (33%). Black horizontal lines denote significant differences (p<0.05). Accuracy increased with any feedback and was best with both forms of feedback. Averaged across all identification tasks. S1: n = 300 (none), 135 (pressure), 165 (aperture), 150 (both). S2: n = 375 (none), 375 (pressure), 375 (aperture), 330 (both).

Artificial sensory feedback significantly improved accuracy in both subjects (Fisher’s Exact Test, p < 0.001). Both subjects performed best when both forms of feedback were provided and worst when no sensory feedback was provided (Fig 2). Feedback type significantly affected the proportion of correct identifications within each 15-trial set (ANOVA, S1: p = 0.003, S2: p < 0.001). In general, when provided with only one form of feedback, both subjects performed better with aperture feedback.

**Fig 3:**
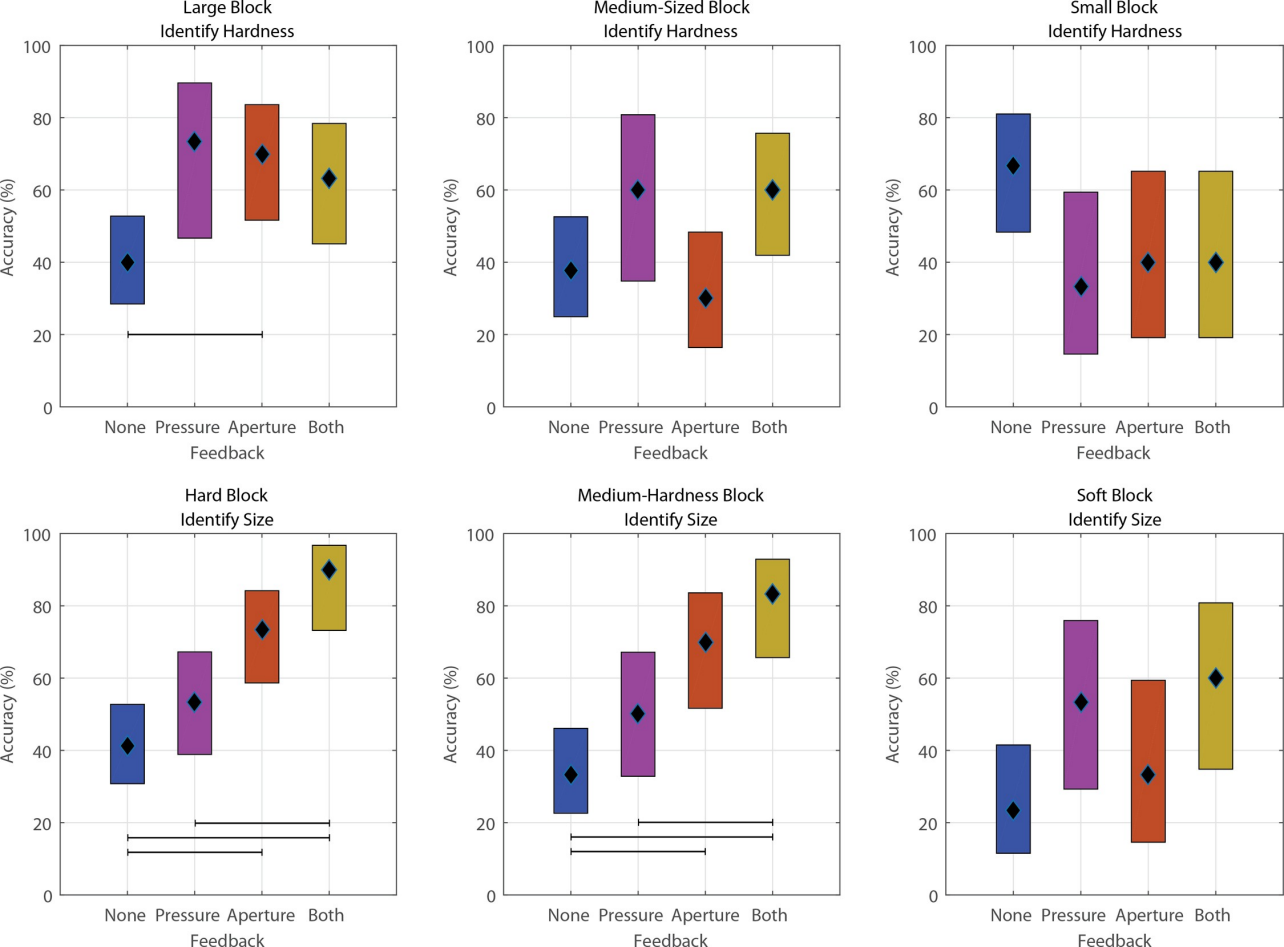
95% confidence interval of the accuracy of block identification for S1. Black diamond denotes the mean. Observations that are significantly different (ANOVA with multiple comparison correction; p<0.05) are distinguished with horizontal bars. In general, S1 performed best when sensory feedback was provided, but the best form of feedback depended on the block identification task. Averaged across all blocks in a particular identification task. Number of observations included in each panel are: top row (left-to-right): 135, 120, 75; bottom row (left-to-right): 195, 150, 75.

**Fig 4:**
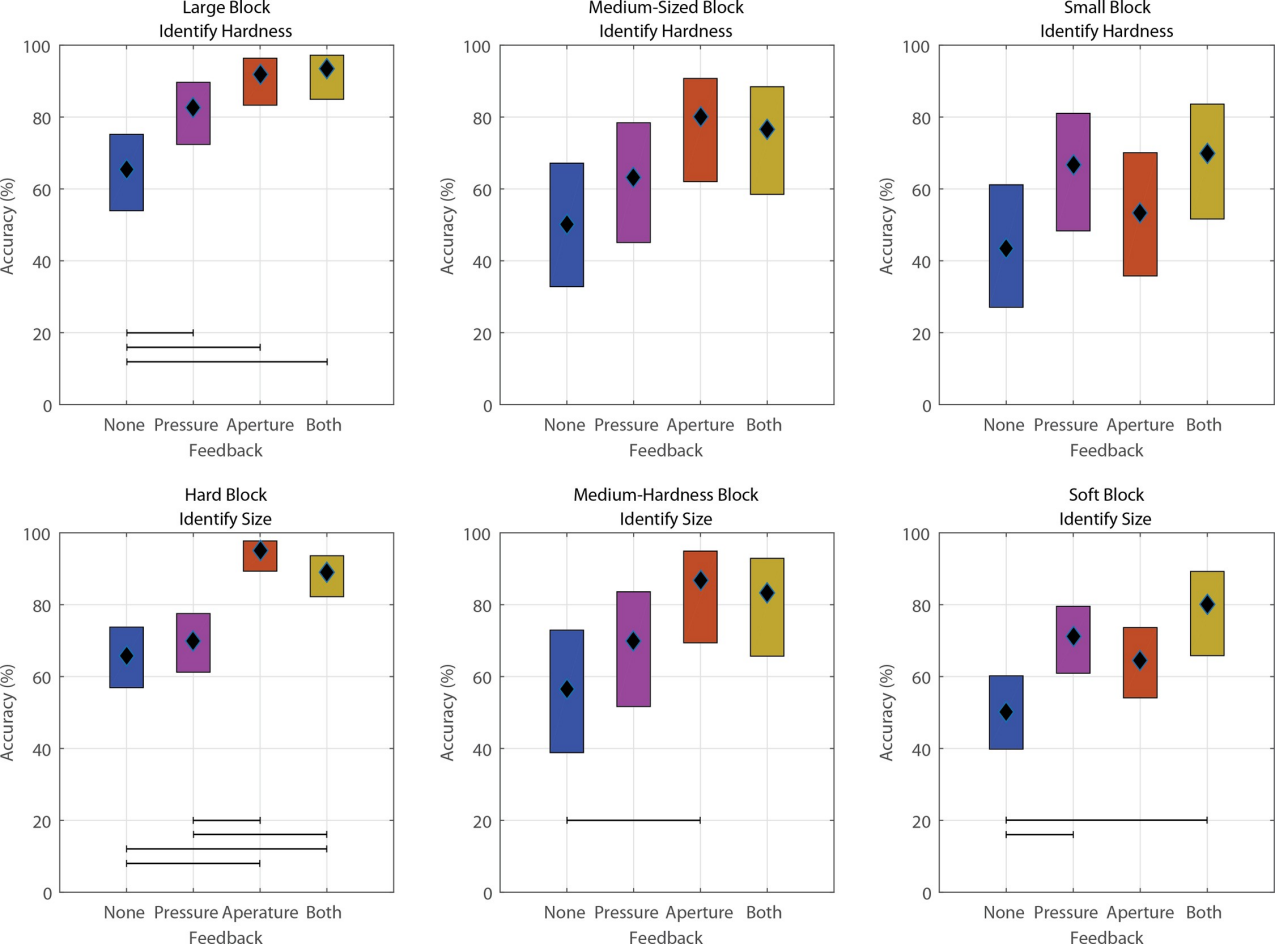
95% confidence interval of the accuracy of block identification for S2. Black diamond denotes the mean. Observations that are significantly different (ANOVA with multiple comparison correction; p<0.05) are distinguished with horizontal bars. In general, S2 performed best when sensory feedback was provided, but the best form of feedback depended on the block identification task. Averaged across all blocks in a particular identification task. Number of observations included in each panel are: top row (left-to-right): 300, 120, 120; bottom row (left-to-right): 480, 120, 315.

In addition to feedback type, accuracy depended on the discrimination task (Figs 3 and 4). As an example of the impact of discrimination task, S1 accurately identified the hard, large block with an accuracy of 62% when choosing hard blocks of varying size, while his accuracy increased to 72% when choosing from large blocks of varying hardness. Conversely, he accurately identified the soft, small block with an average accuracy of 36% when choosing from soft blocks of various size, but his accuracy increased to 64% when choosing from small blocks of varying hardness.

Additionally, different forms of feedback tended to help certain identification tasks more than others. When pressure feedback was provided alone, both subjects exhibited a 7%-8% increase in accuracy during trials in which they determined block compressibility rather than size. When hand aperture feedback was provided, either alone or in combination with pressure feedback, both subjects more accurately identified blocks during trials in which they were determining size rather than compressibility; S1’s accuracy increased by 40% when determining size while S2’s accuracy increased by 2.4%.

In some cases, both subjects performed better than chance (33.33%) even when no stimulus feedback was provided. The performance of both subjects was compared against chance using a one-sample test of proportion. Without stimulus feedback, S1 performed above chance for tests involving small blocks (p<0.001), but did not perform significantly above chance for the other five conditions (0.089≤p≤0.916). S2 performed in an opposite fashion, performing significantly above chance (p≤0.043) on all tests except those involving small blocks (p = 0.165).

### Applied pressure

**Fig 5:**
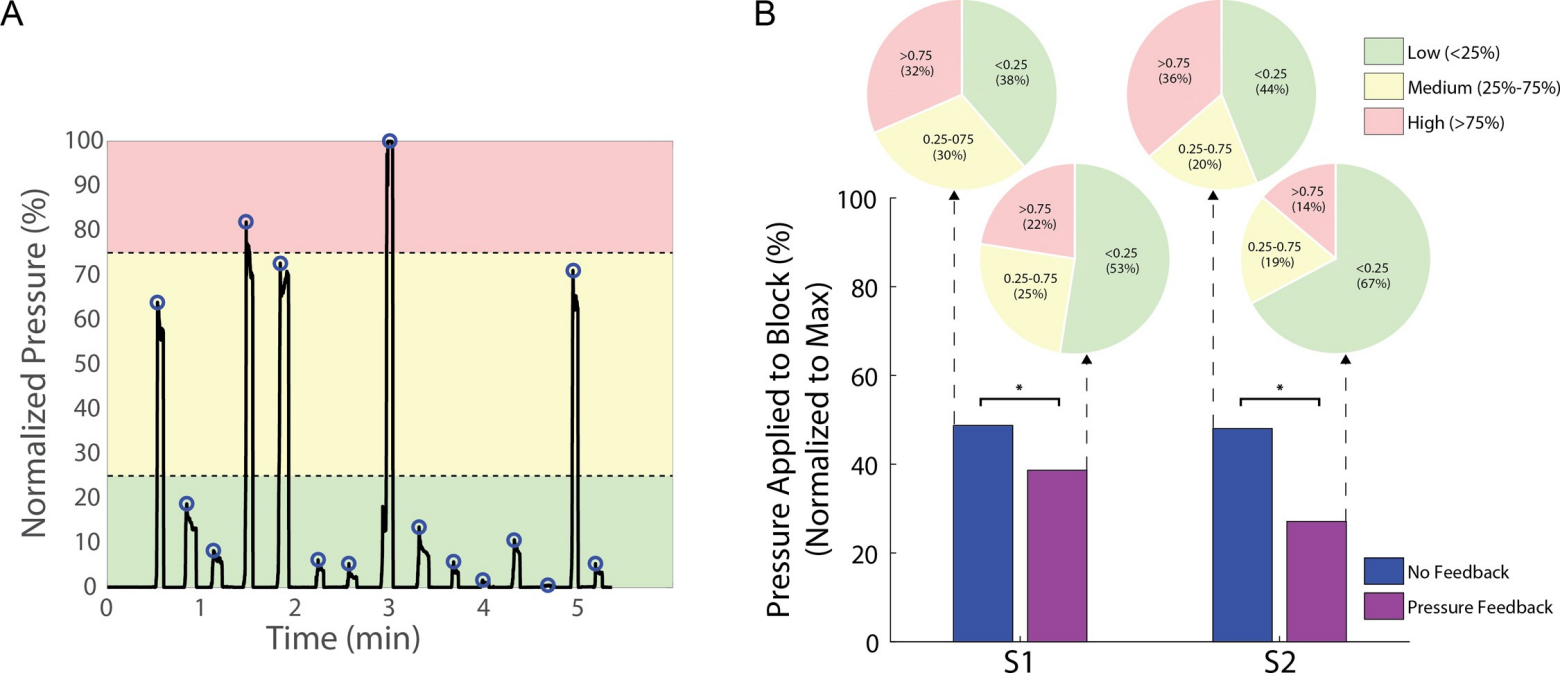
Amount of pressure applied during experiments. A) An example of raw pressure data acquired during a trial. In this trial, the peak pressure for the day was observed during the 8th object presentation around 3 minutes and, thus, normalized to 100%. Colored regions demonstrate how peaks were categorized as “low” (green), “medium” (yellow), or “high” (red) pressure. B) Pie charts: The percentage of normalized applied pressures when binned as low (<25% of the maximum applied pressure), medium (25%-75%) or high (>75%). Bar plots: The average normalized pressure applied over all trials. Both subjects were more likely to apply lower pressure and equally less likely to apply higher pressure when feedback was provided. There was little change in the likelihood of applying moderate pressure. This suggests an overall shift toward application of lower pressure when handling the object.

Sensory feedback type, block size, and block hardness significantly affected the pressure that the subject applied to the block (p < 0.001). S1 exhibited significant interactions between feedback type and block size while S2 exhibited significant interactions between feedback type and block hardness. When sensory feedback was not provided, the applied pressure tended toward a bimodal distribution with a peak at “low” pressures, defined as < 25% of the maximum pressure applied during the foam block tests that day, and a peak at “high” pressures, defined as > 75% of the maximum pressure applied during the foam block tests that day (Fig 5A). Without feedback, both subjects applied low pressures to approximately 40% of foam blocks (green area in Fig 5B) and high pressures to approximately 35% of the foam blocks (red area in Fig 5B). However, when pressure feedback was provided, the applied pressure tended toward a chi-squared distribution with a greater proportion of low-pressure observations. With pressure feedback, both subjects applied low pressures to approximately 60% of foam blocks, representing a 48% increase in the frequency of low pressure application compared to the no feedback condition. At the same time, both subjects applied high pressures to approximately 20% of foam blocks, representing a 47% decrease in the frequency of high pressure application. Because there was very little change in the percentage of observations with “moderate” pressure, it appears that there was a proportional shift of higher pressures into the moderate pressure range and moderate pressures into the lower pressure range. The reduction in normalized pressure (Fig 5B) from 49% to 39% for S1 and from 48% to 27% for S2 was significant (p = 0.008, p < 0.001, respectively).

### Subject confidence

**Fig 6:**
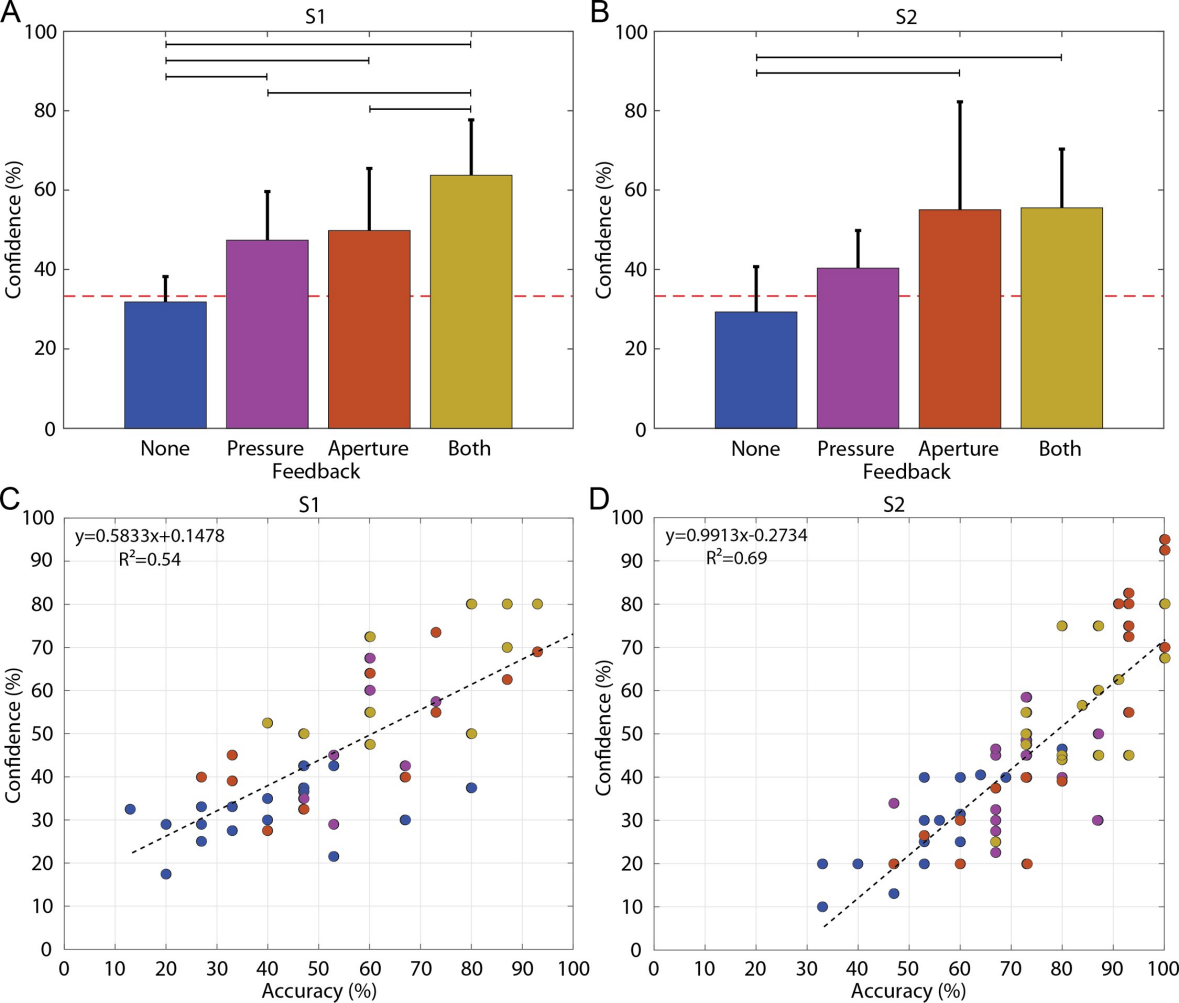
Average confidence levels for S1 (A) and S2 (B) as a function of feedback type. Confidence increased with addition of feedback. There was a significant positive correlation between subject confidence and subject accuracy even though the subject was blinded to results (C and D). Colors indicate the feedback type.

Both subjects were significantly more confident in performing the task with feedback than without feedback (Fig 6A and 6B). For S1, any sensory feedback significantly increased his confidence above the no-feedback condition (p = 0.01 for only pressure, p = 0.001 for only aperture, and p < 0.001 for both pressure and aperture). For S2, confidence was significantly higher with both forms of feedback or with only aperture feedback than without feedback (p < 0.001 for both comparisons). However, there was no significant difference between the only pressure and no feedback conditions (p = 0.255).

Providing both channels of feedback also tended to lead to higher confidence than providing a single channel of feedback. For S1, having both forms of feedback significantly increased confidence over having only one form of feedback (p = 0.019 compared to only pressure, p = 0.041 compared to only aperture; ANOVA with Bonferroni correction). For S2, confidence was nearly significantly higher with both channels of feedback than with pressure only, though this difference was not quite significant (p = 0.069), while confidence with both types of feedback was statistically indistinguishable from proprioception only (p = 1). For S2, confidence with proprioception only was nearly significantly higher than with pressure only (p = 0.069).

Block set also significantly affected subject confidence (S1: p = 0.004; S2: p < 0.001; ANOVA). Confidence was significantly greater for harder blocks than for softer blocks (p < 0.05). On average, confidence was highest when the block set consisted of hard blocks (S1: 52%, S2: 57%) or large blocks (S1: 49%, S2: 54%) and was lowest when the block set consisted of soft blocks (S1: 35%, S2: 32%) or small blocks (S1: 37%, S2: 27%).

S1 reported confidence levels between 18% and 80% (Fig 6C). S2 reported confidence levels between 13% and 95% (Fig 6D). Neither subject was aware of his performance yet both subjects exhibited a confidence that was significantly and positively correlated with OIT accuracy (Pearson’s Correlation; S1: r = 73.6%, p<0.001; S2: r = 83.2%, p<0.001). Subject confidence tended to underestimate actual performance.

## Discussion

In this study, we investigated the effects of tactile and proprioceptive feedback on object discrimination with a UE prosthesis and the relationship between feedback and discrimination task. Further, we characterized the effect of feedback on applied pressure and subject confidence in their abilities. Implanted multichannel nerve cuffs provided sensory feedback that aided prosthesis users in object discrimination tasks. Both subjects performed comparably despite differences in their implant locations, time since amputation, and time since electrode implant.

### Integration of tactile and proprioceptive information

Both subjects tended to identify the size and compliance of deformable objects most accurately when provided with both pressure and aperture sensory feedback via neural stimulation. Both subjects performed worst when provided with no stimulation-elicited sensory feedback. These results demonstrate that subjects were able to perceive the electrical stimulation as relevant sensory information, interpret the sensations relative to their intended prosthesis movements, and make discrimination decisions based on this sensorimotor integration. This finding suggests that sensory input provided through electrical stimulation can be utilized in existing sensorimotor processing pathways. This matches evidence from prior studies on the psychometric properties of sensations elicited by neural stimulation, which suggest that artificial touch is processed in the same way as touch in the intact system [10,25].

Prior studies of able-bodied individuals indicate that integration of both tactile and proprioceptive information is necessary for discrimination of object shape and size [21,22]. Our results align well with these prior findings, since our subjects were most accurate in determining foam block size when provided with both tactile and proprioceptive information. This finding indicates that subjects were able to appropriately integrate these two information streams, even though the information originated from neural stimulation rather than natural mechanoreceptors. The fact that S1 could integrate this artificial proprioceptive and tactile information is surprising, since his prosthesis aperture sensor elicited a tactile percept on his palm rather than a qualitatively proprioceptive percept of hand movement (S1 Fig). This may indicate that the subject was able to learn a remapping of this sensory input during the brief training periods prior to each task.

In able-bodied studies of object discrimination, both tactile and proprioceptive information are also required in determining object compliance, but only if objects are compliant with rigid surfaces [4]. For determining surface compliance, tactile information alone is sufficient. Because the foam blocks utilized in this study had surface compliance (i.e. the surface was not rigid), we expected that tactile (pressure) information would have been most helpful in determining foam block compliance. S1’s results matched this hypothesis, since his performance on compliance discrimination tasks tended to be best with tactile information alone than with either proprioception information alone or both forms of feedback. For S2, object compliance discrimination performance tended to be best with both forms of feedback than with either alone. When determining the compliance of small blocks, his performance with pressure alone was better than with proprioception alone, as expected. However, in the other two compliance discrimination blocks sets, his performance with proprioception alone was better than with pressure alone, contrary to the expected outcome. Although there are several deviations from the expected outcomes based on able-bodied studies, in general the contributions of artificial touch and proprioception to size and compliance discrimination match those of natural touch and proprioception. The differences between the actual and expected trends, however, were not significant.

Based on the data and the subjects’ descriptions of how they made their decisions, the aperture feedback was more useful for identifying the size of stiffer blocks, most likely because the aperture feedback did not change once the fingers contacted the block. When we grouped foam blocks by compressibility (across columns of S2 and S3 Figs) we found that both subjects achieved a higher accuracy when aperture feedback was provided for hard or medium blocks. Conversely, pressure feedback was important for identifying soft blocks (row 3 of S2 and S3 Figs). Pressure feedback resulted in greater accuracy than aperture feedback when identifying the size of soft blocks for both subjects. Consistent with a prior study [23], subjects tended to confuse a block with its nearest neighbor (e.g., small misidentified as medium rather than large) when block misidentifications occurred.

### Restored sensation modifies prosthesis control

Pressure recordings provide insight to how restoring sensation to amputees impacts their prosthetic control. The data show that providing pressure feedback allows the subject to grasp and identify objects without applying maximal pressure. Similar to previous studies utilizing vibrotactile feedback, providing pressure feedback through electrical nerve stimulation yielded applied force reductions of 20% in S1 and 44% in S2 [6,8,26]. This suggests that electrical nerve stimulation will reduce force application and facilitate handling delicate objects.

The reduction in applied force suggests that sensory feedback changed the way that participants controlled their prostheses. Decreased force is direct evidence that the subject stopped closing the hand after perceiving object contact, while without feedback, they continued to contract their residual muscles to drive the prosthesis closed. This finding indicates that artificial sensory information can modify motor control of the prosthetic hand, just as normal tactile feedback can modify control of the intact hand. Previous studies of able-bodied subjects have also shown that specific cutaneous stimuli can provide information about limb position and hand motion that can alter real-time control of hand movements [27,28]. Cessation of active drive of the prosthesis means that less EMG was produced during hand control with sensation, which should delay the onset of fatigue in the user’s arm and extend the duration of prosthetic use.

As with accuracy, there were significant interactions between the feedback type and block presented when analyzing applied pressure. When pressure feedback was provided, both subjects applied the lowest pressure when presented with a large block. The applied pressure increased and became more variable as the block size decreased, regardless of compressibility. Both subjects may have applied the highest pressure to the smallest blocks because the closing speed of the prosthetic hand appeared to increase as hand aperture decreased. The subjects’ myoelectric control schemes are such that the closing speed of the hand depends on the amplitude of the EMG control signal.

### Sensory feedback increases subject confidence

Confidence levels were highest when provided with both forms of sensory feedback. There was a significant positive relationship between accuracy and confidence. Without sensory feedback, confidence was near chance, but was approximately 60% when provided with both forms of feedback. It is possible that the subjects deliberately misreported their confidence based on what they thought we wanted to hear (response bias). However, the confidence was significantly correlated with performance and the subject received no information as to how he performed. Thus, unless the subject deliberately performed poorly with less feedback, the data indicates an improved perception of their abilities to use the prosthesis and improved self-efficacy. Confidence is expected to further increase with practice and visual confirmation during tasks.

### Comparison to prior studies

Our study has several important differences to prior studies of prosthesis function with artificial sensory feedback. In a prior study, we assessed the degree to which neural-stimulation elicited sensory feedback improved detection and manipulation of non-compressible, rigid objects. We found that sensory feedback improved rigid-body object detection and manipulation, increased subject confidence, and improved incorporation of the prosthesis into the user’s sense of self [13]. In this study, we now extend our prior findings to include six 3-object, forced-choice tests using deformable foam blocks, where subjects had to identify properties of the objects rather than presence or absence of objects.

Our study also has several important differences compared to prior studies of object discrimination with artificial sensation[11,23]. First, we used the same blocks for both the compliance and the size discrimination tasks, demonstrating how both object properties can be separately extracted from the same sensory information streams. In contrast, Raspopovic et al. 2014 used different object sets for their two discrimination tasks [11]. Note that in our study, as well as in prior studies [11,23], object size and compliance discrimination were separate tasks. Second, subjects in the present study received multiple sensation locations simultaneously and had to extract the relevant information from these parallel pathways. In our study, subjects received up to four simultaneous locations (when given both tactile and proprioceptive feedback), while in Horch et al. 2011, they received two [23]. In the Raspopovic study, when subjects were asked to determine object compliance, they were given a single sensation location (in their case, the index finger). In their shape discrimination task, they were given two locations (index and pinky), though both were not activated by all objects and there were differences in relative activation time across objects [11]. In our study, we utilized objects (foam blocks) that simultaneously contacted the same set of pressure sensors regardless of block size or compliance. Specifically, we provided sensations simultaneously on the index, middle, and thumb during all trials with tactile feedback, regardless of object properties or discrimination task. Thus, subjects in our study could not use the presence or absence of a sensation location as a cue for object properties. Third, neither prior study investigated the relative contributions of tactile sensation and proprioception to object discrimination performance. Horch et al. 2011 always provided both tactile sensation and proprioception (one location of each), while Raspopovic et al. 2014 only provided tactile sensation in each task [11,23]. Finally, in our study, we compared performance with sensory stimulation to baseline performance with no sensory stimulation, similar to Horch et al. 2011 [23]. Raspopovic et al. 2014 did not have a control condition [11].

### Sensory information availability and congruence

Even though subjects were not provided with sensory stimulation during the “no feedback” conditions and did not receive visual or auditory feedback, they did perform above chance for some tests under this condition. This above chance performance indicates that subjects had access to other sources of information during the task, such as socket vibrations or their internal sense of effort associated with contracting their forearm muscles. During the tests, subjects used their own myoelectric socket, prosthetic hand, and their standard agonist/antagonist control scheme. Therefore, the subjects were very familiar both with the vibratory or other sensations they received through the socket as the hand motors activated and inactivated and with the relationship between prosthetic hand closing speed and muscle effort. Although the subjects indicated that they were guessing during some of the no feedback experiments, at other times they indicated that they tried to rely on the timing between the onset and offset of the motor and that they tried to deliberately control the speed at which the hand was closing. These alternative sources of information were likely sufficient to enable performance above chance levels.

We speculate that, had all possible feedback been removed by disconnecting the prosthetic hand and socket from the arm, the subjects may have performed at chance. However, this condition was not tested in the present study because we sought to maximize real-world applicability of our test, and hand and/or socket disconnection from the limb is not a condition under which the prosthesis would typically be used. In addition, in a prior object discrimination study, a subject was able to perform above chance without stimulation-elicited feedback, despite disconnecting the hand and socket from the subject during the task [23]. Performing above chance without supplemental feedback was also observed in our prior study [13]. Despite a better-than-chance performance without artificial feedback, statistical comparisons between performance with and without sensory stimulation yielded significant differences. Therefore, sensory stimulation is beneficial to prosthetic function, even including referred sensations from the socket and secondary cues typically used by experienced prosthesis wearers.

When setting up each subject’s neural stimulation for the task, efforts were made to match the location and modality of elicited sensory percepts with the information transduced by the prosthesis sensors (S1 Fig). However, in one case, for S1’s aperture sensor, this was not possible. S1 does not have any proprioceptive sensations elicited by neural stimulation, so the aperture sensor on his prosthesis was mapped to a tactile percept on his palm. This resulted in differences between the subjects in terms of the congruence of the sensory percepts and the transduced information. Although the trends in performance on the tasks were largely consistent between subjects, there were a few differences between the subjects. The differences in performance between subjects may have resulted, at least in part, from this difference in modal and spatial congruency for the aperture sensor. More congruent sensations may be more useful or more easily integrated with multimodal sensory information. This study did not explicitly examine modal congruency, so these observations require follow-up studies to test this hypothesis.

Interestingly, S2’s performance across both the compliance and size discrimination tasks suggests that he had greater confidence in or relied more on the proprioceptive information than the tactile information. He was marginally better at determining object size when provided with proprioceptive information alone than with both forms of feedback, and tended to be better at determining object size when provided with both forms of feedback than when provided with tactile information alone. This greater weighting of proprioception was also observed for this subject in a prior study of the impact of sensory feedback in rigid object discriminations [13]. The differences in weighting between the two forms of feedback for this subject may have been due to differences in the relative intensity or modal congruence of the percepts. While we attempted to match the perceived intensity of the elicited percepts across all sensory inputs (both tactile and proprioceptive), it is possible that some inputs were perceived to be more intense than others. The subject may then have relied more heavily on sensory channels that were perceived to be more intense, because they were more readily noticeable or accessible. In addition, the modal congruence of the stimulation-elicited percepts to the subject’s expectations may have influenced the relative weightings of the information. S2’s aperture sensor elicited a proprioceptive sensation of hand closing that was modally-matched to his expectations, whereas the pressure sensors elicited tactile sensations that may not have matched the subject’s expectations for tactile modality. Follow-up studies could explore the role of sensation intensity and modal congruence in decision making and functional prosthesis performance.

Subjects in this study perceived multiple locations of sensation simultaneously: tactile sensation on the first, second, and third digits; and a proprioceptive sensation of hand closing or a proprioceptive substitute on the palm. Although the prosthetic used in the tasks was a single degree of freedom and the second and third digits were mechanically linked, we chose to provide tactile sensation for both the second and third digits rather than a single digit. This was because we believed that having sensation locations that were congruent with the movements of the prosthesis were important for subject interpretation of the sensations while performing tasks. For example, if we had provided sensation only on the second digit but not the third, subjects may have become confused by the lack of visual-tactile congruence when contacting the block with both the second and third digits, but only receiving sensory stimulation associated with the second digit. Although subjects were blindfolded during the trials, they received visual information during the training period. The training period enabled participants to become familiar with the blocks in the block set for that trial set and with the sensory feedback provided. We do not know whether the results would be different if we had provided sensation on only the second or only the third digit or if there were differential information clues between the different sensors that the user included in the decision process.

We did not compare object discrimination performance between the prosthesis and the intact hand. Since preliminary tests of intact hand performance demonstrated 100% accuracy, these comparisons would not have been meaningful. The fact that the subjects performed perfectly with their intact hand was not surprising because neither had tactile deficits. In addition, comparisons between the intact hand and the prosthetic would have been inappropriate because of differences in the information available to the participant. Because the blocks of different compliances also differed in surface properties, intact hand object discriminations may have been governed by texture cues. In contrast, the prosthesis sensors could not transduce texture, as each only transduced normal force at a single location. In addition, the prosthesis was a single degree-of-freedom hand and thus could only contact the objects using a single grasp, whereas the intact hand would not be limited in hand posture or grasp. Additional grasps or hand postures would have fundamentally altered the information available to the subject, resulting in different tasks between hands.

### Foam block identification as a test for restored sensation

An objective in this study was to assess the suitability of this test battery in assessing the functional capabilities of prosthesis users following sensory restoration. We found that accuracy was as low as 13% without any feedback and could be as high as 100% with both pressure and aperture feedback. Accuracy depended both on feedback type and discrimination task. Importantly, the identification of size of the soft or medium compressibility blocks or the compliance of the small or medium-sized blocks rarely resulted in 100% accuracy, even with both forms of sensory feedback. This is not surprising, given that subjects were using a single DOF prosthetic hand with standard agonist/antagonist control, only four channels of sensory feedback, and had limited practice with the test during each session. This lack of a performance ceiling suggests that our test battery would be an attractive method to measure the impact of increased sensation locations, the impact of restored sensation in combination with advanced prosthetic controllers or dexterous hands, and the effect of learning how to use sensation over time. In future studies, the foam block set could be modified so that there is no visual indicator of compliance, but it would be difficult to make objects of various size appear visually the same. These object discrimination tasks could also be used to understand the impact of visual and/or cognitive distractors, and these studies could be performed without blindfolding the subject.

## Conclusions

Subjects could identify block size and hardness when provided with sensory feedback in which they felt a sensation at their phantom fingertips collocated with the application of pressure to the prosthesis. Accuracy and confidence were highest when provided with sensory feedback, and depended both on feedback type and discrimination task. Providing sensory feedback also reduced the pressures applied to the blocks when making the identifications. This demonstrates the potential for improving object recognition and confidence by adding sensory feedback to prostheses.

We believe this test battery provides an effective means to assess the impact of sensory restoration and the relative contribution of different forms of feedback (tactile vs. kinesthetic) within the neurorehabilitation field. The 9-choose-3 foam test battery provided a wide range of testing conditions. The trends between the two subjects were typically consistent, suggesting that this 9-block assay should be used in future sensory restoration studies and should be standardized by collecting normative data on additional amputee and able-bodied subjects.

## Supporting information

S1 Fig Prosthetic hand sensor locations and corresponding perceived sensory locations evoked by neural stimulation. A) A subject wearing a prosthetic hand, with externally-attached sensors, grasping a foam block. B) Prosthesis sensor locations (left) and perceived sensory percept locations (right). Pressure sensors on the thumb, index, and middle fingers made contact with the block while it was grasped and transduced normal force. The aperture sensor deflected along with the prosthesis’ closing motion. The perceived sensory locations displayed are the average percept location for stimulation contacts typically associated with each prosthesis sensor for each subject. Subjects drew the location and extent of the sensory percept on hand diagrams, and these images were digitized and processed using MATLAB (Mathworks, Natick, MA). Sensory locations are overlaid such that regions of higher opacity were reported more frequently. The black, double-ended arrow indicates a proprioceptive sensation of hand movement, where the index and middle fingers were perceived to move towards the thumb. Note that for subject 1, the aperture sensor corresponded to a tactile percept on the palm. Note that for the aperture sensor for subject 2, the hand region highlighted in brown is the region involved in the movement sensation.
(TIFF)

S2 Fig Mean accuracy rate for each block type presented to S1. Sensory feedback always increased identification accuracy except for the soft, small-sized blocks.
(TIF)

S3 Fig Mean accuracy rate for each block type presented to S2. Sensory feedback always increased identification accuracy.
(TIF)
