# Supplementary figures and images for: Artificial tactile and proprioceptive feedback improves performance and confidence on object identification tasks
Source: PLoS One. 2018 Dec 5;13(12):e0207659. doi: 10.1371/journal.pone.0207659 (PMC6281191; doi:10.1371/journal.pone.0207659)

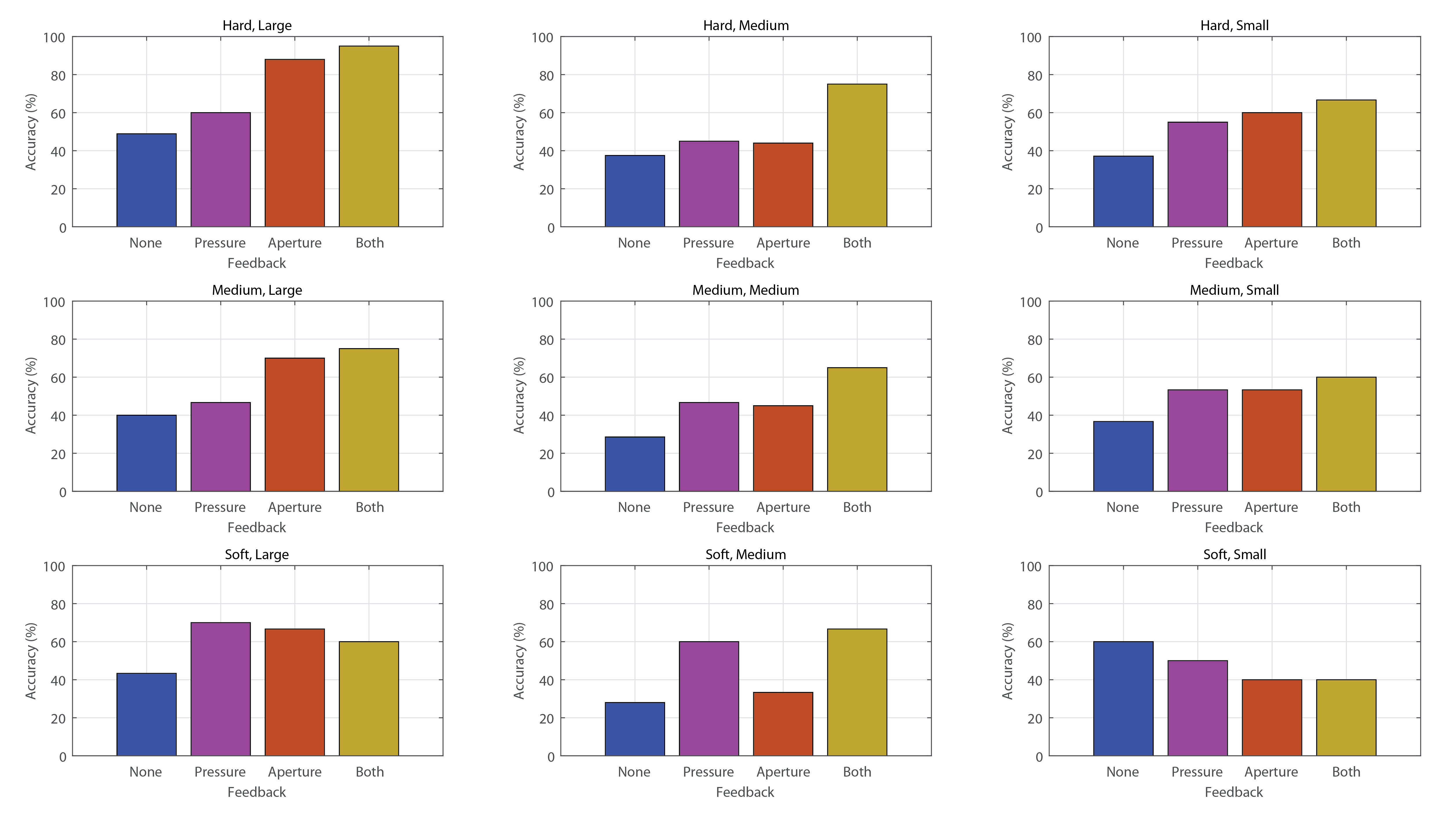

Supplement: S2 Fig — Sensory feedback always increased identification accuracy except for the soft, small-sized blocks. (TIF) [file pone.0207659.s002.tif]

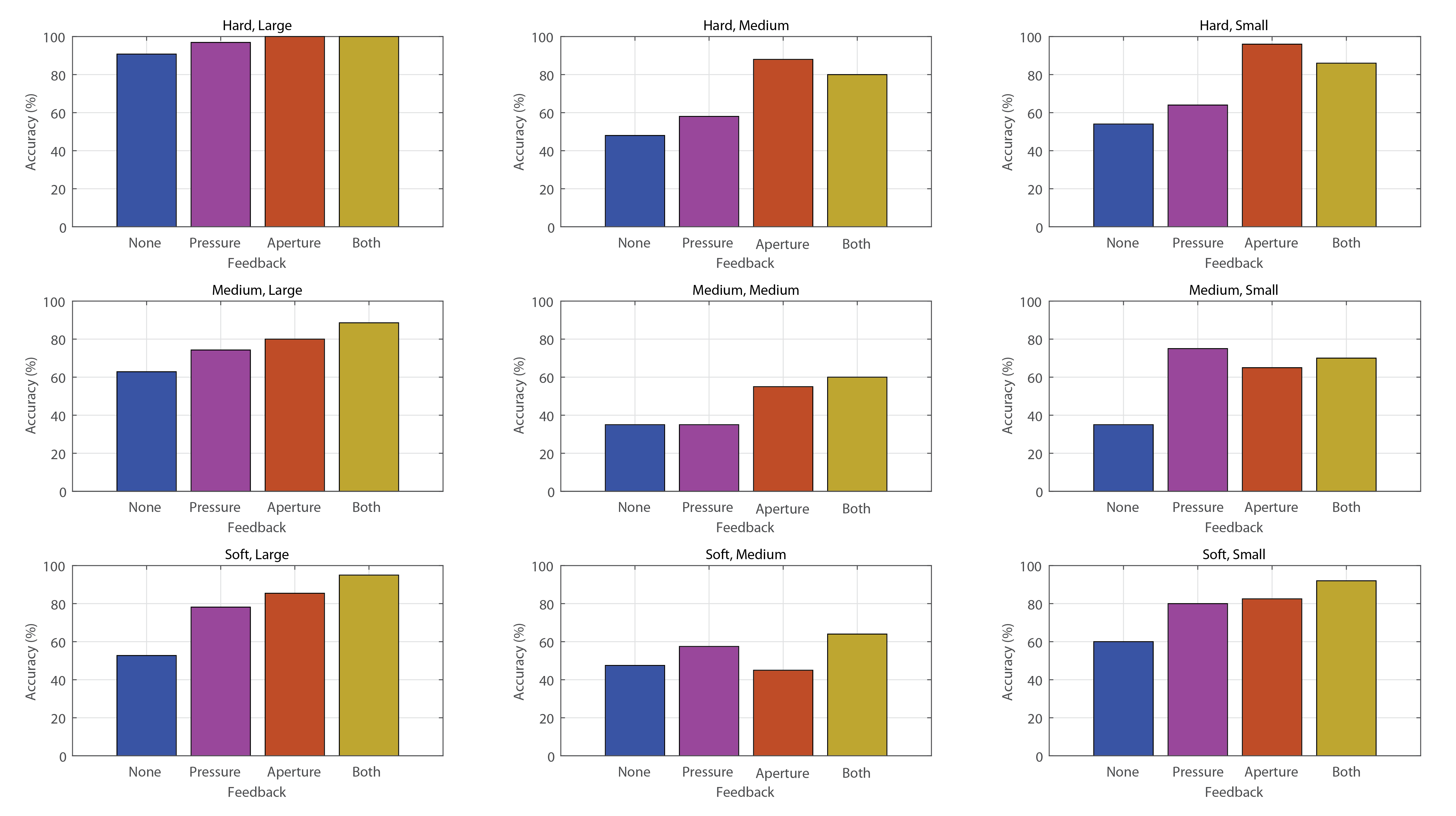

Supplement: S3 Fig — Sensory feedback always increased identification accuracy. (TIF) [file pone.0207659.s003.tif]
